# Pathological complete response after afatinib treatment of stage IV oligometastatic adenocarcinoma of the lung: the role of pulmonary surgery

**DOI:** 10.1186/s40792-019-0741-3

**Published:** 2019-11-12

**Authors:** Ping-Chung Tsai, Yi-Chen Yeh, Chien-Sheng Huang, Chao-Hua Chiu

**Affiliations:** 1 Division of Thoracic Surgery, Department of Surgery, Taipei Veterans General Hospital, 201, Section 2, Shih-Pai Road, Taipei, Taiwan; 2 Institute of Clinical Medicine, School of Medicine, National Yang-Ming University, Taipei, Taiwan; 3 Department of Pathology, Taipei Veterans General Hospital, Taipei, Taiwan; 4 Institute of Clinical Medicine, School of Medicine, National Yang-Ming University, Taipei, Taiwan; 5 Division of Thoracic Oncology, Department of Chest Medicine, Taipei Veterans General Hospital, Taipei, Taiwan

**Keywords:** Oligometastases, Pathologic complete response, Stereotactic radiotherapy, Immune checkpoint inhibitors, Tyrosine kinase inhibitors

## Abstract

**Background:**

Some oligometastatic lung cancer patients, after induction systemic chemotherapy or tyrosine kinases inhibitor treatment, followed by aggressive radical consolidative treatment, have improved overall survival. Unfortunately, clinical criteria cannot assess such patients.

**Case presentation:**

We hereby reported the case of a 55-year-old female with lower back pain and bilateral lower leg numbness for months and who had an osteolytic bone lesion over the third lumbar vertebra. In February 2017, a third lumbar vertebra biopsy showed metastatic adenocarcinoma, compatible with lung origin (thyroid transcription factor-1 positive [TTF-1], L858R mutation positive). Complete imaging of the right lower lobe (RLL) showed a spiculated mass of about 3.4 × 2.2 cm, and a trans-bronchoscopic lung biopsy revealed non-small cell carcinoma of lung origin (positive for TTF-1 and negative for p40). Tentative diagnosis was RLL adenocarcinoma, cT2aN0M1b, with bone metastasis at L3. The epidermal growth factor receptor-tyrosine kinase inhibitor afatinib was prescribed beginning April 2017. A November 2018 follow-up CT scan showed regression in the RLL lung mass. A whole-body positron emission tomography-computed tomography showed RLL lung nodule with faint uptake and mildly increased uptake in the L3 vertebra. After providing informed consent, the patient received uniportal video-assisted thoracoscopic RLL lobectomy and radical mediastinal lymph node dissection on December 25, 2018. The final pathology report was fibrotic scar with no residual tumor cells, compatible with post-treatment status, ypT0N0. Curative intent radiotherapy was also applied to the L3 vertebra after the operation. The patient is still alive for more than 32 months after initially diagnosed with metastatic lung adenocarcinoma.

**Conclusions:**

Our case provides additional data to support that tissue assessment through primary lung tumor resection after systemic treatment of oligometastic lung cancer by minimally invasive surgery can reveal the treatment effect and potentially provide a surrogate endpoint in further clinical trials.

## Introduction

Surgical resection is the primary treatment for patients with early-stage non-small cell lung cancer (NSCLC), but not those with advanced-stage or metastatic NSCLC, although radical consolidative treatment (RCT), including original pulmonary resection of oligometastatic NSCLC, improves survival [1, 2]. The objective response rate to epidermal growth factor receptor (EGFR) mutations-tyrosine kinase inhibitors (TKI) for advanced NSCLCs is ~ 60%, but few achieve a complete response. No correlation exists between pathological complete response (pCR) and clinical and pathological factors, including “Response Evaluation Criteria in Solid Tumors” (RECIST) criteria. Furthermore, computed tomography (CT) RECIST response does not correlate with major pathologic response [3], and positron emission tomography (PET) Response Criteria in Solid Tumors (PERCIST 1.0) is still under clinical assessment [4]. Accurate non-operative assessment of pCR is currently impossible. Surgical resection can identify pCR and may help stratify patients for additive therapy. pCR after induction therapy is an independent prognostic factor in patients with locally advanced NSCLC, but rarely report [5] in the patients with oligometastatic NSCLC.

## Case presentation

**Fig. 1:**
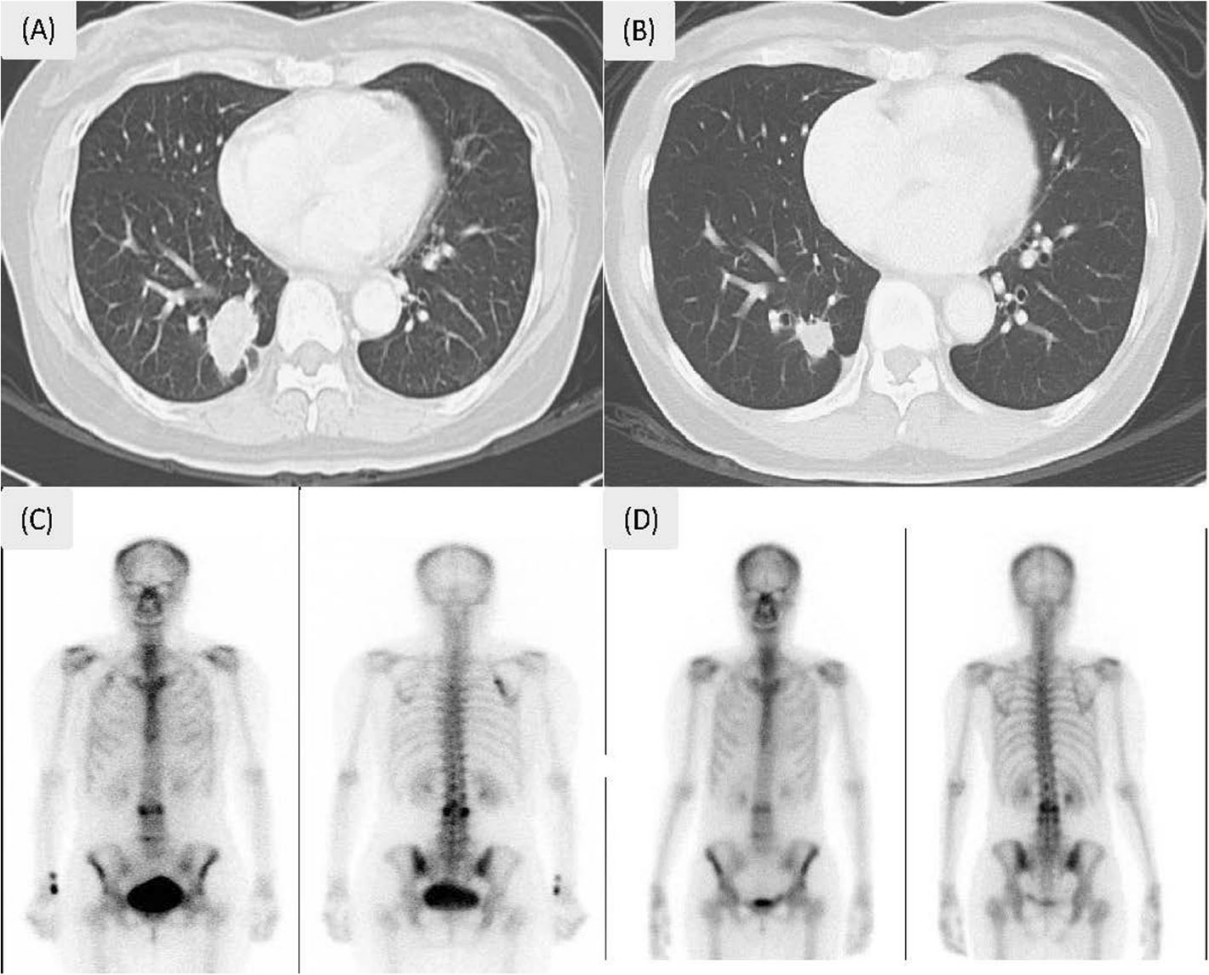
Patient scans. **a** Chest computed tomography scan: pre-tyrosine kinase inhibitors (TKI) therapy in March 2017. **b** Post-TKI therapy and pre-surgery in November 2018; right lower lobe lung mass regressed but noticeable. **c** Whole body bone scan: pre-TKI in March 2017. **d** During TKI therapy in July 2018

**Fig. 2:**
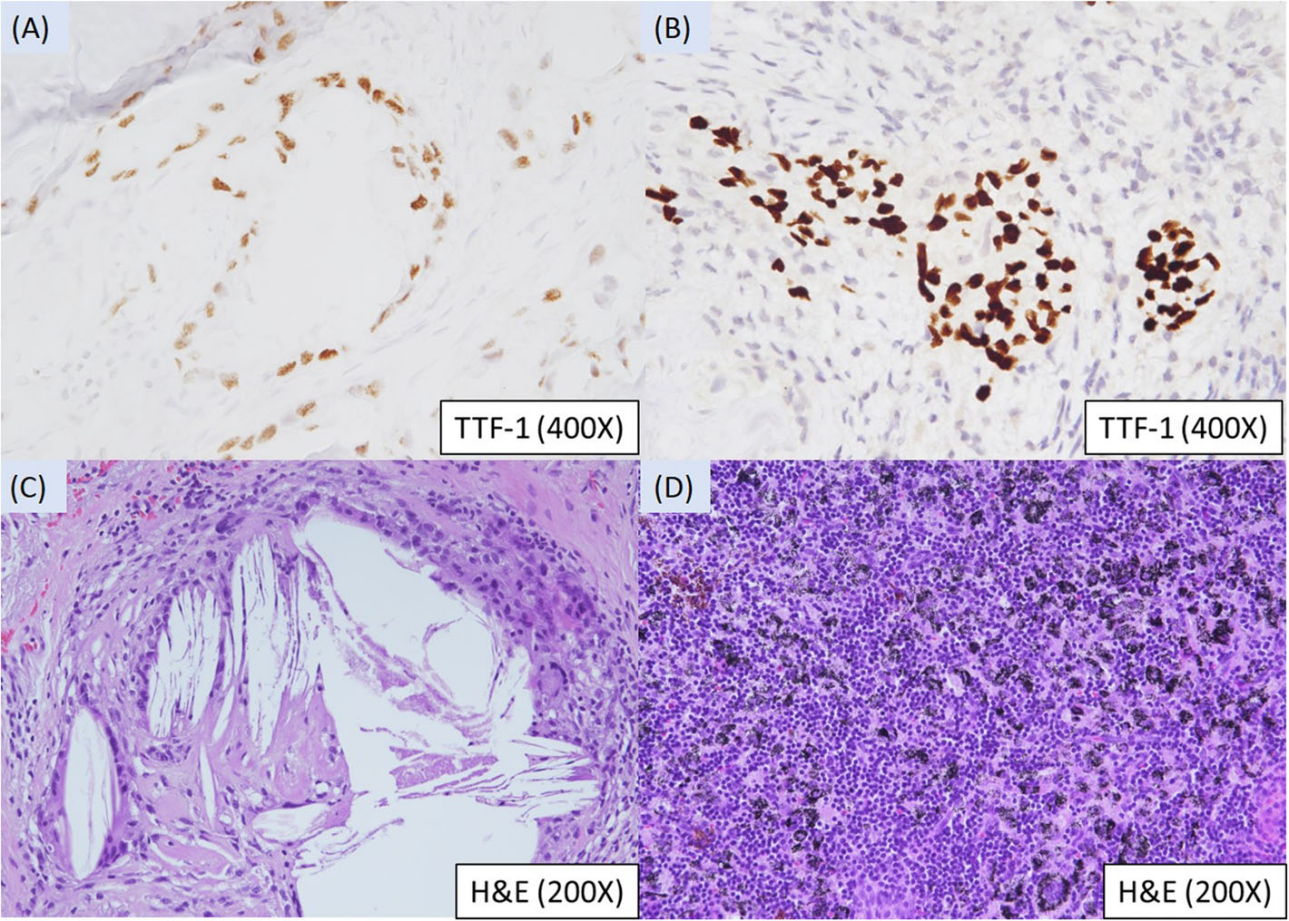
Biopsies and staining results. **a** Biopsy from the third lumbar vertebra, TTF-1(+). **b** Bronchoscopic biopsy from the RB10, TTF-1(+). **c** Lung adenocarcinoma with complete tumor regression with cholesterol crystals and multinucleate giant cells. Hematoxylin and eosin stains. **d** Lymph node: anthracosis only

A 55-year-old female with lower back pain and bilateral lower leg numbness for months had an osteolytic bone lesion over the third lumbar vertebra (L3). In February 2017, an L3 vertebra biopsy showed metastatic adenocarcinoma, compatible with lung origin (TTF-1 positive, L858R mutation positive). Complete imaging of the right lower lobe (RLL) showed a spiculated mass about 3.4 × 2.2 cm with pleural traction. A trans-bronchoscopic lung biopsy revealed non-small cell carcinoma of lung origin (positive for TTF-1 and negative for p40). Tentative diagnosis was RLL adenocarcinoma, cT2aN0M1b, with bone metastasis at L3. Routine use of serum tumor markers for monitoring of the tumor burden revealed a higher carcinoembryonic antigen (CEA) level that was still within the normal range (4.9 < 5.0 ng/ml), and an abnormal level of CA-125 (37.1; normal range 35.0 U/ml). The EGFR-TKI afatinib was prescribed beginning April 2017. However, side effects (severe diarrhea, poor appetite, and body weight loss) required dosage reduction to 40 mg every other day. A November 2018 CT scan showed regression in the RLL lung mass, and the density of lumbar bone lesion became unapparent in the whole-body bone scan compared with scans from 2017 (Fig. 1). A whole-body PET-CT showed RLL lung nodule with faint uptake and mildly increased uptake in the L3 vertebra. A series of declining serum CEA levels down to 1.1 ng/ml were noted after starting afatinib treatment with no signs of elevation prior to surgical intervention. Serum CA-125 level down to 16.8 U/ml (< 35) was also noted after starting afatinib treatment 3 months later, and there was no further follow-up due to stable of disease. For alternative treatment evaluation, the patient went to the surgical outpatient department and asked for possible surgical intervention of the primary tumor. After providing signed informed consent, the patient received uniportal video-assisted thoracoscopic RLL lobectomy and radical mediastinal lymph node dissection on December 25, 2018. The final pathology report was a fibrotic scar with no residual tumor cells, compatible with post-treatment status, ypT0N0 (Fig. 2). Curative intent radiotherapy with 30 Gy/12 fraction was also applied to the L3 vertebra after the operation. The patient is still alive for more than 32 months after initially diagnosed with metastatic lung adenocarcinoma.

## Discussion

Defined as the absence of tumor cells in resected specimens after neoadjuvant treatment (ypT0N0), pCR is an important prognostic factor in managing locally advanced NSCLC [6] but rarely discussed in oligometastic NSCLC. Recently, Gomez et al. reported a phase 2 randomized trial and demonstrated that oligometastic NSCLC patients responded to induction systemic treatment followed by treatment with RCT, and the overall survival achieved was 41.2 months [1]. Iyengar et al. reported another phase 2 randomized trial and showed benefit of RCT by radiotherapy prior to maintenance chemotherapy in patients with limited metastatic NSCLC, nearly tripling progress-free survival over maintenance chemotherapy alone [2]. Even with improved treatment of oligometastic NSCLC, to our knowledge, no clinical report has indicated pCR in oligometastatic lung adenocarcinoma after afatinib treatment.

Interestingly, the post-afatinib treatment chest CT scan showed regression in the RLL lung mass, and whole-body PET-CT showed RLL lung nodule with only faint uptake in our case. Arrieta et al. also reported that 19 (54%) patients achieved a complete response assessed by PET-CT before and after RCT in oligometastatic NSCLC, who have significantly longer median overall survival compared to those who had a non-complete response (NR vs. 27.4, *p* = 0.011), rendering PET-CT an important potential prognostic marker [7]. Although only with post-treatment PET-CT which showed faint uptake of the primary lung tumor, our case report can echo the possibility as an imaging marker of PET-CT in oligometastic NSCLC management. On the other hand, similar to a Dutch study, patients with a documented PET had a superior survival compared to those without (11.6 vs.8.2 months) [8]; upstaging effects of PET-CT (Will Rogers phenomenon) should also be considered.

From a surgical standpoint, the effect of primary lung resection in patients with metastatic disease had not been well assessed [9]. Meanwhile, the superiority of surgical resection over radiologic ablation therapy as a RCT for primary lung cancer in oligometastatic lung cancer remains unclear, although minimally invasive surgery and stereotactic radiotherapy both have gained interest with increasing availability [10].

Although phase 2 randomized trials have demonstrated survival benefits for oligometastatic lung cancers when the disease is stable or regression occurs after systemic induction treatment followed by treatment with RCT [1, 2], decisions regarding the timing of surgical intervention for primary lung tumor are still difficult to make. Accordingly, surgical intervention for primary lung tumor, including metastatic RCT (lumbar bone in our case), is recommended as early as possible in patients who present with stable disease or regression after systemic induction treatment and before maintenance therapy. The benefit of this strategy is to allow the disease to declare itself, and patients who develop early and disseminated progression are not considered for local RCT. However, several therapeutic sequences should still be considered and may provide benefits to oligometastatic lung cancer patients [5], for example, up-front metastatic RCT first, followed by systemic therapy and lung surgery. The majority of patients who present with oligometastatic brain metastasis reported in the literature underwent brain radical treatment (craniotomy or gamma knife radiosurgery) prior to systemic and lung surgery, mainly due to the presence of neurologic symptoms.

Furthermore, immune checkpoint inhibitors (ICI) have revolutionized the treatment strategies in advanced NSCLC. Although radiotherapy and ICI act synergistically, radiotherapy is thought to be the preferred RCT technique. The same comment is raised: how to evaluate the treatment effect? Recently, Bott et al. reported two patients with pCR but still showed stable disease radiographically after ICI treatment for patients with resectable NSCLC (pseudo-progression phenomenon) [11]. In brief, even in the era of advances in molecular target medicine and ICI, tissue assessment through primary lung tumor resection after systemic treatment of lung cancer is still the only strategy to reveal treatment effects currently.

The other critical issue is how long EGFR-mutated lung adenocarcinoma achieves a long-lasting response to afatinib after treatment discontinuation. Rare cases have been reported that were maintained for years through complete remission with EGFR-TKI treatment [12]. Nevertheless, tumor flares have been reported in patients who discontinued an EGFR-TKI because of disease progression or inability to tolerate treatment, as well as in patients with progression while on EGFR-TKI who have responded after treatment [13]. Whether afatinib can be discontinued in the treatment of lung cancer when pCR is achieved over the long term after surgical resection is still a controversial issue. In our case, after discussion with the patient, systemic therapy using afatinib is continuously prescribed every other day as maintenance therapy after primary lung resection.

## Conclusion

To conclude, here, we report the first case of pCR to afatinib treatment in a patient with synchronous oligometastic lung adenocarcinoma who presented with histology-proven bone metastasis. Our case provides additional data to support that tissue assessment through primary lung tumor resection after systemic treatment of oligometastic lung cancer by minimally invasive surgery can reveal treatment effects and potentially provide a surrogate endpoint in further clinical trials.

## Data Availability

Data sharing is not applicable to this article, as no datasets were generated or analyzed during the current study.

## Abbreviations

CEA: Carcinoembryonic antigen
CT: Computed tomography
EGFR: Epidermal growth factor receptor
ICI: Immune checkpoint inhibitors
L3: The third lumbar vertebra
NSCLC: Non-small cell lung cancer
pCR: Pathological complete response
PET: Positron emission tomography
RCT: Radical consolidative treatment
RECIST: Response Evaluation Criteria in Solid Tumors
RLL: Right lower lobe
TKI: Tyrosine kinase inhibitors
TTF1: Thyroid transcription factor-1
